# Increased Amount of Polyunsaturated Fatty Acids in the Intestinal Contents of Patients with Morbid Obesity

**DOI:** 10.1007/s11695-023-06518-1

**Published:** 2023-02-25

**Authors:** Agata Janczy, Michal Szymanski, Marta Stankiewicz, Lukasz Kaska, Krzysztof Waleron, Ewa Stelmanska, Tomasz Sledzinski, Adriana Mika

**Affiliations:** 1 Department of Food Commodity ScienceDepartment of Food Commodity Science, Faculty of Health Sciences with the Institute of Maritime and Tropical Medicine, Medical University of Gdansk, ul. Debinki 7, 80-211 Gdansk, Poland; 2 Department of General, Endocrine, and Transplant Surgery, Faculty of Medicine, Medical University of Gdansk, ul. Smoluchowskiego 17, 80-214 Gdansk, Poland; 3 Department of Clinical Nutrition, Faculty of Health Sciences with the Institute of Maritime and Tropical Medicine, Medical University of Gdansk, ul. Debinki 7, 80-211 Gdansk, Poland; 4 Department of Pharmaceutical Microbiology, Faculty of Pharmacy, Medical University of Gdansk, Al. Gen. J. Hallera 107, 80-416 Gdansk, Poland; 5 Department of Biochemistry, Faculty of Medicine, Medical University of Gdansk, ul. Debinki 1, 80-211 Gdansk, Poland; 6 Department of Pharmaceutical Biochemistry, Faculty of Pharmacy, Medical University of Gdansk, ul. Debinki 1, 80-211 Gdansk, Poland; 7 Department of Environmental Analysis, Faculty of Chemistry, University of Gdansk, ul. Wita Stwosza 63, 80-308 Gdansk, Poland

**Keywords:** Obesity, Microbiota, Diet, Polyunsaturated fatty acids, Fat metabolism

## Abstract

**Introduction:**

Obesity is associated with disturbed gut microbiota homeostasis that translates into altered intestinal and blood metabolite profiles. The long-chain fatty acid (LCFA) may be absorbed in the intestine, but until now, their composition in intestinal contents of patients with obesity has not been studied. The aim of the present study was to verify whether obesity is related to any changes in fecal LCFA content and whether intestinal LCFA content may be associated with the health status of patients with obesity.

**Methods:**

The fatty acid composition has been studied in stool samples obtained from 26 patients with morbid obesity and 25 lean subjects by gas chromatography–mass spectrometry. The dietary habits were assessed using the Food Frequency Questionnaire (FFQ-6).

**Results:**

Our results show for the first time that lean subjects and patients with obesity differ in their stool LCFA profiles. The levels of most n-3 polyunsaturated fatty acids (PUFAs) and n-6 PUFAs were significantly higher in fecal samples from people with obesity than in those from lean controls.

**Conclusions:**

Based on the current knowledge, we have defined three hypotheses that may explain proving the cause-and-effect relationships observed differences in fecal LCFA profiles between patients with obesity and lean subjects. They may be related to alterations in fat digestion and/or LCFA absorption and diet. However, proving the cause-and-effect relationships requires further research.

**Graphical Abstract:**

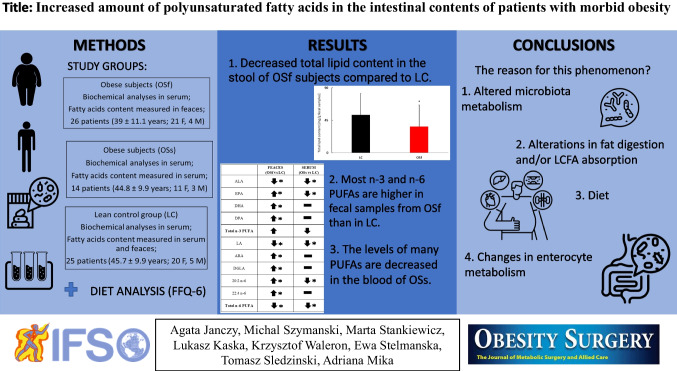

**Supplementary Information:**

The online version contains supplementary material available at 10.1007/s11695-023-06518-1.

## Introduction

Obesity is a common disease with high morbidity and mortality rates. Although the hereditary component of the disease is strong, the sources of the obesity pandemic cannot be explained solely by genetic factors [1]. The main cause of this phenomenon is believed to be unfavorable lifestyle changes, especially a decrease in physical activity and increased consumption of processed and high-energy food products [2]. An important element in the pathogenesis of overweight and obesity is disturbed gut microbiota homeostasis [3–5]. The obesity-related dysbiosis results in the formation of increased amounts of lipopolysaccharide (LPS), which then enters the bloodstream, where it acts as the main cause of metabolic endotoxemia and induces the production of proinflammatory cytokines [5]. It is worth noting that the lipid composition of the diet is of great importance in the development of endotoxemia. A diet high in saturated fatty acids (SFAs) has been shown to contribute to the development of endotoxemia, while a Mediterranean diet containing larger amounts of polyunsaturated fatty acids (PUFAs) and oils containing n-3 PUFAs reduces metabolic endotoxemia [6, 7]. PUFAs and SFAs can differentially modulate the action of Toll-like receptor 4 (TLR4), which activates proinflammatory pathways. Since the ligand for this receptor is a fragment of LPS-containing SFAs, their replacement with PUFAs leads to a reduction in LPS toxicity [8].

Our previous studies revealed many changes in the profiles of serum long-chain fatty acids (LCFAs, defined as fatty acids with 12 or more carbons in their chains) in patients with severe obesity and in those who had undergone bariatric surgeries [9–15]. There is a great deal of published research on obesity-related changes in the concentration of short-chain fatty acids (SCFAs) in the intestinal contents [16–18]. In contrast, to the best of our knowledge, there are no published data on the level of LCFAs in the stool of patients with obesity. The intestines are a site of lipid digestion and absorption; thus, the content and absorption of LCFAs in the intestines may impact their content in blood and various tissues. We recognize that fecal fat content comes mainly from bacteria and from undigested dietary fats, while the rest are from epithelial cells that are shed [19]. We hypothesize that LCFA stool content differs between lean and obese subjects and that this difference may contribute to metabolic alterations in subjects with obesity. The aim of the present study was to verify whether obesity is related to any changes in fecal LCFA content and whether intestinal LCFA content may be associated with the health status of patients with obesity.

## Materials and Methods

### Patients and Control Subjects

The main study group included 26 patients with obesity (mean age 39 ± 11.1 years; mean BMI 41.0 ± 5.65 kg/m^2^, 21 females, 4 males) who qualified for bariatric surgery (BS) at the Centre of Obesity and Metabolic Diseases (COMD) of our medical university, from whom fecal samples were collected — the OSf group (OSf — subjects with obesity included in the analysis of feces). The selection of participants for the study group was based on the following inclusion criteria: age over 18 years, and qualification for bariatric surgery. The exclusion criteria were as follows: previous bariatric surgery, neurodegenerative disease, type-2 diabetes mellitus, specific or nonspecific bowel diseases, medical history of partial bowel resection, transplantation of microbiota, long-term antibiotic therapy (e.g., in the course of Lyme disease), current probiotic therapy, and antibiotic therapy less than 3 months before inclusion in the study. Six patients presented with metabolic syndrome. The patients with obesity collected their own samples before beginning a low-calorie diet in preparation for surgery.

The lean control group (LC) consisted of 25 subjects (mean age 45.7 ± 9.9 years; 20 females, 5 males). The criteria for including subjects in the control group were a normal body mass index (at least 18.5 but less than 24.9 kg/m^2^), age over 18 years, and good health status assessed on the basis of medical history (absence of clinical evidence for endocrine (including diabetes), cardiac, hepatic, cancer, or renal diseases) and routine biochemical tests. Other exclusion criteria were the same as in the group of patients with obesity.

The subjects from the control group agreed to come for blood donation on the day of fecal sample collection. Unfortunately, since the fecal samples from patients with obesity were collected at home by the patients themselves, then frozen for storage and transferred to the laboratory within 1–3 days, it was not possible to take blood at the same time. Thus, to evaluate the changes in the levels of serum LCFAs in patients with severe obesity, we included an additional group of patients with obesity, from whom blood for serum preparation was collected during the first visit to the clinic, before the patients began a specialized diet in preparation for bariatric surgery; this group, designated the OSs group (OSs–subjects with obesity included in the analysis of serum), included 14 patients (mean age 44.8 ± 9.9 years; mean BMI 37.6 ± 1.88 kg/m^2^, 11 females and 3 males). Six patients presented with metabolic syndrome. The exclusion criteria were the same as in the OSf group. The division of the study subjects into groups is shown in Fig. S1.

The study was performed in agreement with the principles of the World Medical Association’s Declaration of Helsinki and was approved by the Local Bioethics Committee at our medical university (decision no. NKBBN/512/2020). All participants gave written informed consent for inclusion in the study.

Standard laboratory markers were examined by the Central Clinical Laboratory of our medical university.

### Food Questionnaire

All participants’ dietary habits were assessed using the Food Frequency Questionnaire (FFQ-6), a semiquantitative tool that has been validated for the Polish population and allows researchers to assess the usual frequency (times/person/day) and quality of 62 groups of products consumed in the last 12 months [20]. The details of the FFQ questionnaire are presented in supplementary materials (Suppl. File 1).

### Sample Collection

The participants themselves, including both patients with obesity (OSf) and lean subjects, collected the stool samples into sterile containers with a plastic spatula in accordance with the instructions provided to them. Samples were taken from 6 different places to maintain the diversity and homogeneity of the material, and then the samples obtained from all study subjects were immediately frozen at − 20 °C and delivered to the laboratory within 3 days. There, the samples were stored at − 80 °C until analysis [21].

After an overnight fast, blood samples were collected from patients with severe obesity (OSs group) and LC subjects into tubes without anticoagulant, kept at room temperature until it was clotted, and then centrifuged at 3000 × *g* to obtain serum, which was stored at − 80 °C until analysis.

### Lipid Analysis

According to the method of Folch et al. [22], total lipids were extracted from 300 μL serum, the homogenate was obtained from 250 mg fecal samples, and additionally, the bacterial fraction was isolated from 500 mg of a fecal sample. The bacterial fraction has been isolated from fecal samples of the nonobese subject using the method described by Hevia et al. [23]. Briefly, diluted fecal homogenate was centrifuged with 40% and 30% iodixanol in an ultracentrifuge with a swing-out rotor SW 28 (Beckman) for 1.5 h at 13,200 × g (10,000 rpm) at 10 °C. After centrifugation, the bacterial fraction separated from the debris was collected by gentle pipetting. Fatty acid levels were assayed by gas chromatography–mass spectrometry (GC–MS) using a QP-2010 SE apparatus (Shimadzu, Kyoto, Japan) as previously described [24].

### Statistical Analysis

All statistical analyses were carried out using Statistica 13.3 (StatSoft, Kraków, Poland). All the data are presented as the means ± standard deviations (SDs). The outliers among the data were identified using Grubbs’ test. To assess whether the quantitative variable was from a population with a normal distribution, the W Shapiro–Wilk test was used. This test confirmed that the analyzed data came from normally distributed populations. The statistical significance of differences between subjects with morbid obesity and lean controls was evaluated with Student’s *t* test for parametric data, and the Mann–Whitney Rank Sum test for non-parametric data. To evaluate the relationship between the variables, the Spearman test was used to calculate the correlation coefficients. The differences were considered significant at *p* < 0.05.

## Results

The subjects from the OSf group presented with significantly higher body mass index (BMI) values, HOMA index, and serum concentrations of glucose, insulin, and C-reactive protein (CRP) than the LC subjects. The serum concentration of high-density lipoprotein cholesterol (HDL-C) was significantly lower in the OSf group than in the LC group (Table 1).

**Table 1:** Selected biochemical and anthropometric characteristics in the study groups

Parameter	LC	OSf
Age (years)	45.7 ± 9.93	39.0 ± 11.1^#^
BMI (kg/m^2^)	23.2 ± 2.33	41.0 ± 5.65^#^
TC (mg/dl)	198 ± 31.4	201 ± 40.7
HDL-C (mg/dl)	59.4 ± 12.1	50.0 ± 11.6*
LDL-C (mg/dl)	116 ± 31.3	121 ± 31.4
TAG (mg/dl)	113 ± 67.5	134 ± 75.2
ALT (U/l)	ND	25.0 ± 11.9
AST (U/l)	ND	36.1 ± 29.4
Glucose (mg/dl)	87.2 ± 6.26	94.4 ± 9.51*
Insulin (mU/ml)	7.14 ± 2.56	16.2 ± 10.3^#^
HOMA-IR	1.54 ± 0.55	3.78 ± 2.33^#^
CRP (mg/l)	1.56 ± 1.11	7.61 ± 5.36*
Albumin (g/l)	39.3 ± 1.98	41.2 ± 2.81*
Interleukin 6 (pg/ml)	ND	3.32 ± 0.89

Data are presented as mean ± SD. *LC*, lean controls; *OSf*, subjects with obesity (the group included in the analysis of feces); *BMI*, body mass index; *TC*, total cholesterol; *HDL-C*, high-density cholesterol; *LDL-C*, low-density cholesterol; *TAG*, triacylglycerols; *ALT*, alanine aminotransferase; *AST*, aspartate aminotransferase; *CRP*, C-reactive protein; *ND*, not determined. *Significant difference compared with healthy controls at *p* < 0.05; ^#^Significant difference compared with healthy controls at *p* < 0.001

### Stool Fatty Acid Composition in Subjects with Obesity

**Fig. 1:**
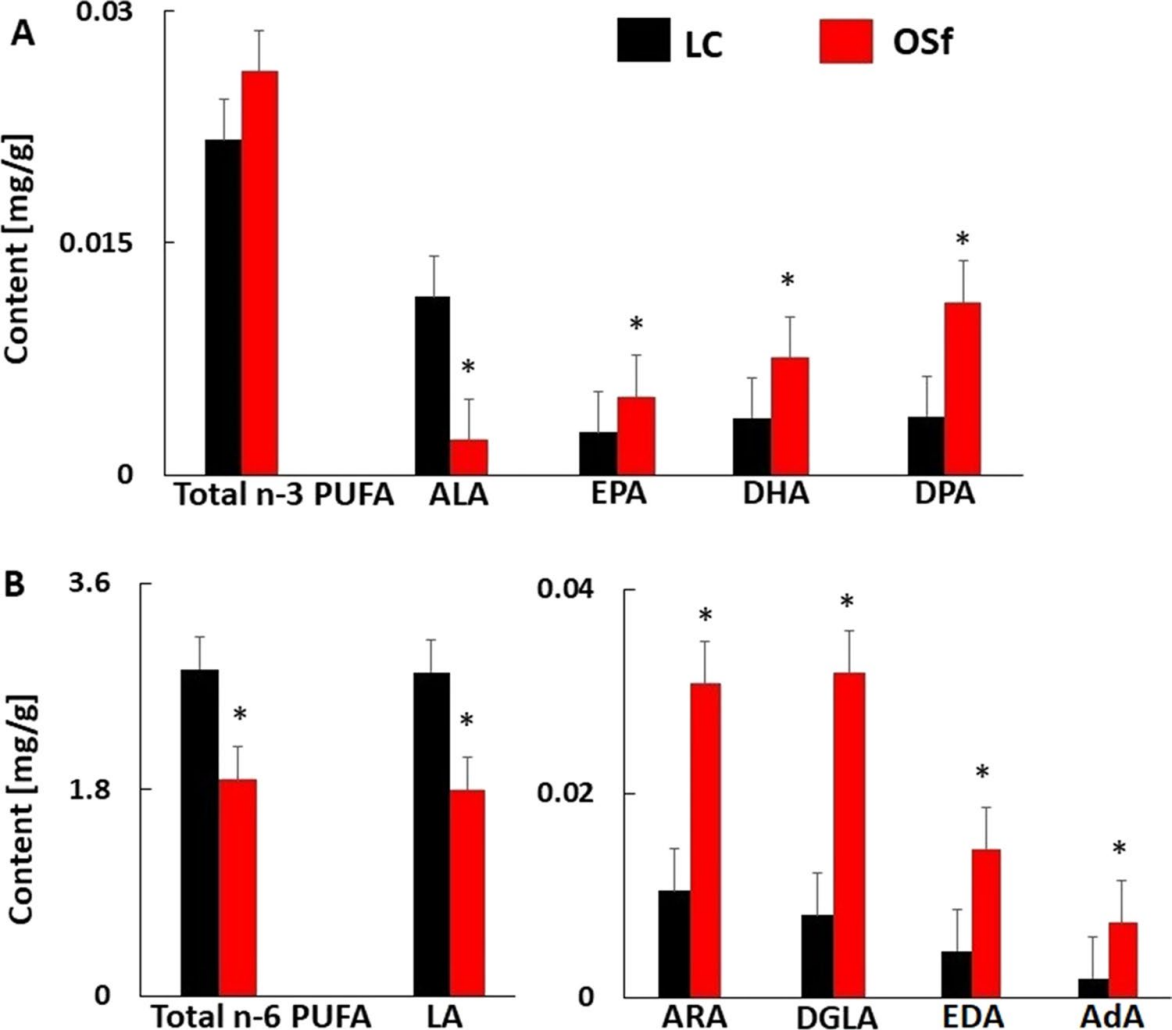
Comparison of the fecal n-3 (**A**) and n-6 (**B**) PUFA content (mg/g) in study groups. Data are presented as mean ± SD. LC, lean controls; OSf, subjects with obesity (group included in analysis of faces). The significance is as follows: ALA *p* = 0.003, EPA *p* = 0.036, DHA *p* = 0.031, DPA *p* = 0.018, Total n-3 PUFA *p* = 0.243, LA *p* = 0.041, ARA *p* = 0.005, DGLA *p* = 0.010, EDA *p* = 0.007, adrenic acid (AdA; 22:4 n-6) *p* = 0.004, Total n-6 PUFA *p* = 0.005

Analysis of LCFA composition in stool samples showed that the total content of n-3 PUFA in the OSf group (patients with severe obesity) did not differ significantly from the LC group (Fig. 1); however, the differences in longer n-3 PUFAs were statistically significant. In the LCFA group, the content of α-linolenic acid (ALA; 18:3 n-3) was significantly lower in the stool of OSf patients compared to the LC group. All other longer n-3 PUFAs were more abundant in the stool of patients with obesity, at a statistically significant level (Fig. 1).

The total content of n-6 PUFA and linoleic acid (LA; 18:2 n-6) in the feces of OSf patients was statistically lower than in the LC group (Fig. 1). In contrast, all longer n-6 PUFAs were significantly more abundant in the stool of OSf patients than in lean subjects.

The results of the analysis of the whole LCFA profile in stool also showed differences in the content of some individual LCFAs from other groups. Since our study is the first to characterize the LCFA profile in fecal samples of subjects with obesity, and some LCFA are not present in the serum of patients (e.g., some BCFA), we have decided to present the main groups of LCFA in Table 2, and the full LCFA profile, including over 50 individual molecules, in Supplementary Table 1.

**Table 2:** Fatty acids content (mg/g) in feces from lean controls and subjects with obesity

Fatty acids	LC	OSf	*p*
Total ECFA	9.40 ± 3.19	8.20 ± 2.26	0.073
Total OCFA	0.41 ± 0.21	0.34 ± 0.17	0.098
Total *anteiso* BCFA	0.26 ± 0.15	0.22 ± 0.12	0.149
Total *iso* BCFA	0.24 ± 0.14	0.14 ± 0.08	0.004
Total tri-methyl -BCFA	0.01 ± 0.01	0.00 ± 0.00	0.032
Total BCFA	0.56 ± 0.28	0.39 ± 0.20	0.010
Total SFA	10.37 ± 3.53	8.93 ± 2.46	0.056
Total MUFA	6.74 ± 2.11	4.66 ± 1.47	0.001
Total Cyclopropane FA	0.03 ± 0.02	0.02 ± 0.01	0.036
Total n-3 PUFA	0.02 ± 0.02	0.03 ± 0.03	0.243
Total n-6 PUFA	2.85 ± 2.31	1.89 ± 1.54	0.050
Total LCFA	2.88 ± 2.31	1.93 ± 1.53	0.051

Data are presented as mean ± SD. The significant difference compared with healthy controls at *p* < 0.05. *ECFA*, even-chain saturated fatty acids; *OCFA*, odd-chain fatty acids; *BCFA*, branched-chain fatty acids; *SFA*, saturated fatty acids; *MUFA*, monounsaturated fatty acids; *Cyclopropane FA*, cyclopropane fatty acids; *PUFA*, polyunsaturated fatty acids, *LCFA*, long-chain fatty acids

Additionally, due to a statistically significant age difference between the LC and OSf group (see Table 1), we excluded subjects with the outstanding age to check if it may affect observed differences in FAs profile. However, the changes in FAs profile were similar in smaller groups with comparable ages (see Suppl. Tables 2 and 3).

The analysis of bacterial fraction isolated from stool revealed that bacterial cells contain low contents of LA, ARA, and ALA, whereas other PUFA were in trace amounts or below the level of detection (supplementary Fig. 2).

**Fig. 2:**
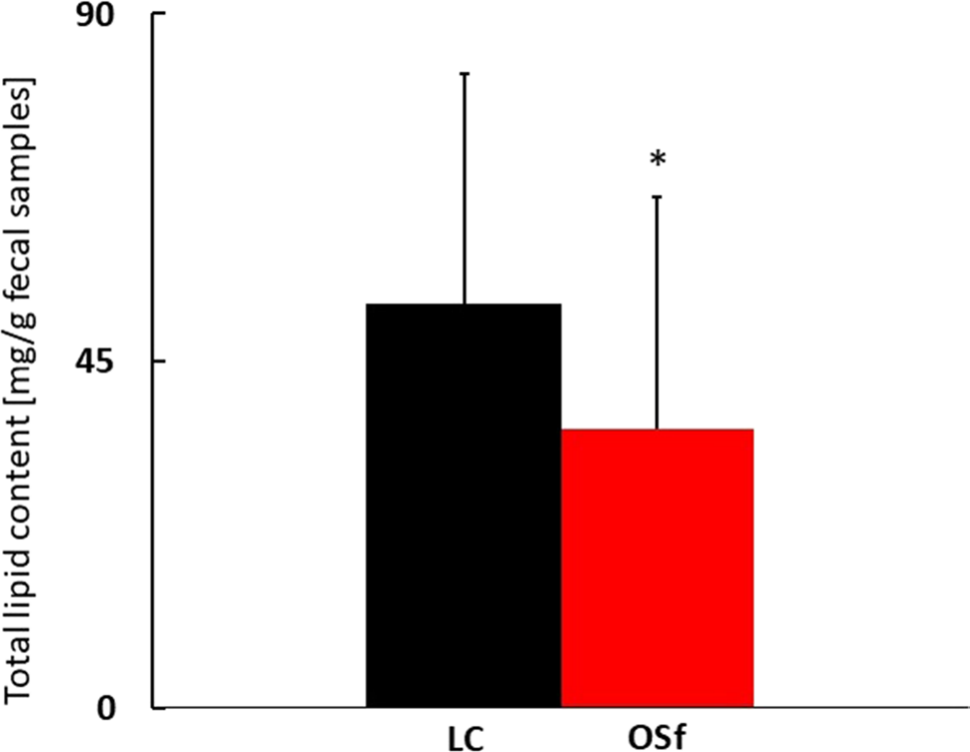
Comparison of the total lipid content in fecal samples (mg/g) in study groups. Data are presented as mean ± SD. LC, lean controls; OSf, subjects with obesity (group included in analysis of faces) (*p* = 0.02)

Since most of the analyzed FA groups were decreased in the stool of subjects with obesity or tended to decrease, moreover, the total LCFA levels were about 30% lower in the OSf group, and this difference was almost statistically significant (*p* = 0.051), we assumed that it can be a result of lower lipid concentration in the stool of subjects with obesity. We have calculated the total content of stool lipids in both groups based on the amounts of the extracted lipids and sample mass. Indeed, the total lipid content in the stool of people with morbid obesity (OSf) turned out to be lower than that of lean subjects (*p* = 0.02) (Fig. 2).

The compositions of the study subjects’ diets were assessed based on the FFQ-6 questionnaires, which were completed by the subjects from the OSf and LC groups. Statistical analysis showed no significant differences in the types of consumed food products between the two study groups, which indicates that there were no major differences in dietary composition between the subjects from the two groups. An attempt was also made to correlate the consumption of selected dietary fat sources with individual n-3 and n-6 PUFAs in the stool of the OSf group. Spearman correlation analysis showed a few correlations (Supplementary Table 4).

### Serum Fatty Acid Composition in Subjects with Severe Obesity

Unfortunately, we were not able to collect stool and blood from OSf patients at the same time. However, we collected blood from another group of subjects with severe obesity (OSs) and compared their serum LCFA composition with that of lean volunteers to determine whether the changes in the LCFA profile in stool samples translated into serum LCFA profiles. The characteristics of the OSs group were generally similar to those of the OSf group (please compare Table 1 and supplementary Table 5).

**Fig. 3:**
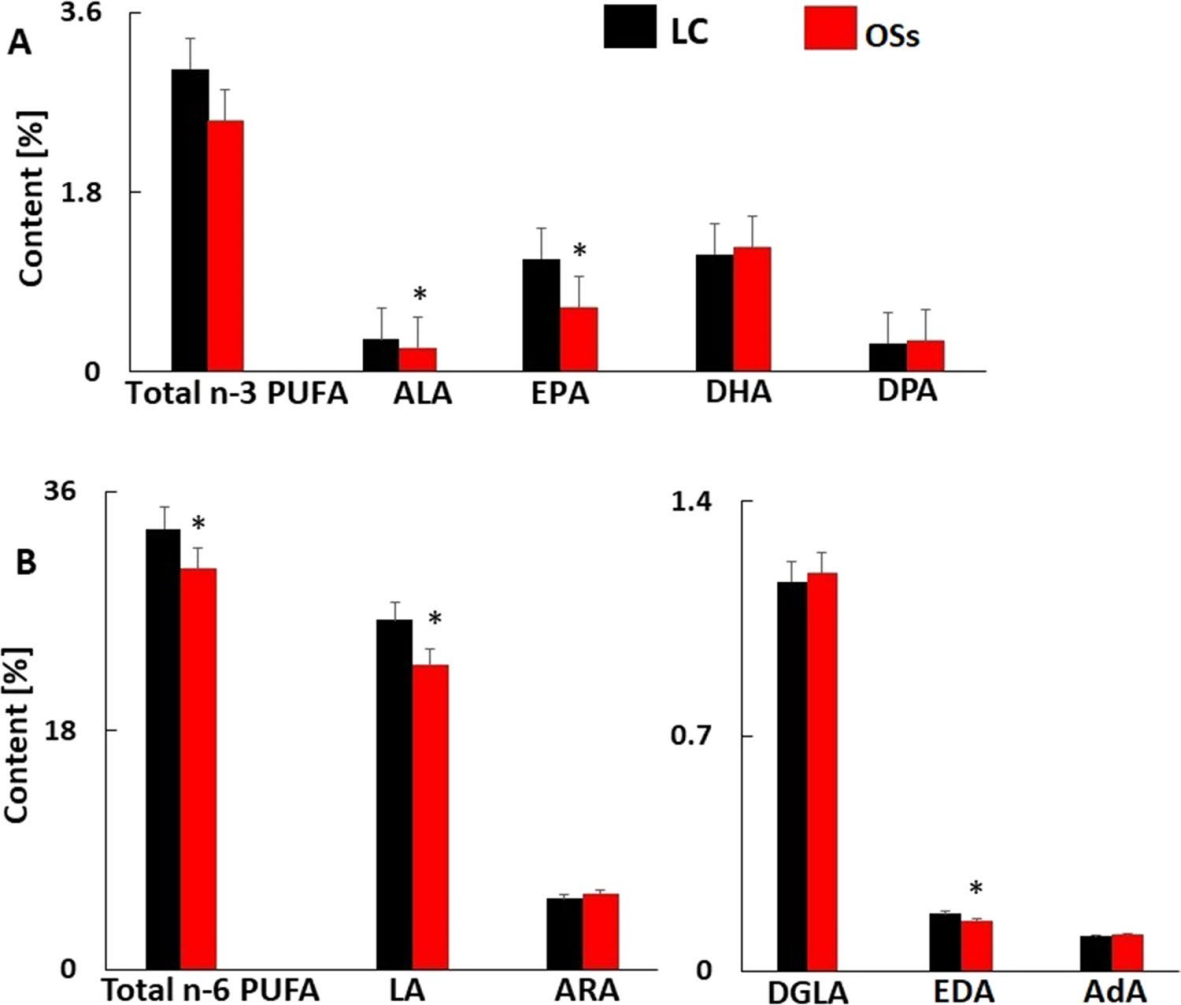
Comparison of the serum n-3 (**A**) and n-6 (**B**) PUFA content (%) in study groups. Data are presented as mean ± SD. The significance is as follows: ALA *p* = 0.001, EPA *p* = 0.047, DHA *p* = 0.658, DPA n-3 *p* = 0.354, Total n-3 PUFA *p* = 0.079, LA *p* = 0.013, ARA *p* = 0.430, DGLA *p* = 0.782, EDA *p* = 0.041, AdA *p* = 0.637, Total n-6 PUFA *p* = 0.045

As shown in Fig. 3, the differences in serum n-3 PUFA and n-6 PUFA profiles between patients with obesity and lean subjects were unlike those observed in the fecal samples. A trend toward reduced total n-3 PUFA content was also observed in the serum of OSs patients compared to the LC group (Fig. 3), while in OSf fecal samples, the content of n-3 PUFA tended to be higher than observed in the LC group (Fig. 1). The EPA and eicosadienoic acid (EDA; 20:2 n-6) contents were significantly lower in the serum samples of the OSs group than in those of the LC group (*p* = 0.047, *p* = 0.041 respectively, Fig. 3), whereas in the stool samples, the contents of these LCFAs were higher in OSf patients than in controls (Fig. 1). By contrast, significantly reduced levels of ALA, LA, and total n-6 PUFAs were observed in the serum samples of OSs patients compared to the control group (Fig. 3), which was similar to the results seen in feces (Fig. 1). In summary, the individual PUFA levels in the serum of subjects with obesity were lower than or did not differ from those of lean subjects (see Supplementary Table 6). A comparison of the total fatty acid profiles in serum samples from the OSs and LC groups is presented in Supplementary Table 6. The trends and differences in stool and serum PUFA composition between lean and subjects with obesity are summarized and compared in Supplementary Table 7.

## Discussion

To the best of our knowledge, the results presented in this study are the first to show the LCFA composition in the stool of subjects with obesity. We determined the levels of over 50 different LCFAs and compared them with the results obtained from stool samples of lean controls (Table 2). Based on the current knowledge, we have defined three hypotheses that may explain the observed differences in fecal LCFA profiles between patients with obesity and lean subjects. They may be related to (a) altered microbiota composition and metabolism, (b) alterations in fat digestion and/or absorption in subjects with obesity, and (c) diet.

### Altered microbiota composition and metabolism

The most pronounced differences between patients with obesity and lean subjects’ LCFA profiles were found in the PUFA group. The total n-3 PUFA content in the stool tended to be higher in the OSf group than in the LC group, and the only ALA, the shortest of the detected n-3 PUFAs, with only an 18-carbon chain was lower in the stool of subjects with obesity. Similarly, the total content of n-6 PUFA and LA in the stool was significantly lower in the study group; all other, longer n-6 PUFAs were significantly more abundant in fecal samples of patients with obesity than lean subjects. Overall, our results show that the stool content of PUFAs with carbon chain lengths greater than 18 was higher in people with obesity than in lean people. All these longer PUFAs can be synthesized from ALA and LA by fatty acid elongases and desaturases. This might suggest that the intestinal microbiota of subjects with obesity may have increased activity of the abovementioned enzymes; however, the analysis of bacterial fraction isolated from the stool sample showed that human intestinal bacteria contain only a small amount of LA, ARA, and ALA (about ten times lower than their total content in stool); trace amounts of dihomo-gamma-linolenic acid acid (DGLA; 20:3 n-6) and other PUFA were not detected. This makes the hypothesis of a bacterial origin of intestinal PUFA unlikely.

### Altered Fat Digestion and Absorption

Another reason for the observed increase in the abundance of PUFAs in the feces of patients with obesity may be the differences in fat digestion and absorption between patients with obesity and lean people. PUFAs in the form of monoacylglycerol are absorbed from the intestinal lumen into enterocytes by diffusion, and free-form PUFAs are taken up by specific transporters, namely CD36 and FATPs [25, 26]. It has been shown that the composition of dietary lipids can influence the density of FA transporters in the plasma membrane [27]. Thus, changes in fat digestion and absorption may be added to potential reasons for alterations in intestinal FA content. This speculation is also supported by the comparison of LCFA profiles in the stool and serum of patients with morbid obesity. Generally, the serum levels of individual n-3 PUFAs and n-6 PUFAs were lower in OS patients than in lean controls. These serum FA profiles of OSs patients are compatible with the results from our other studies [10, 12] and other authors’ studies [28, 29]. The observed phenomenon might be much more complex and can be correlated with disturbances of intestinal microbiota. Supplementary Table 5 presents a comparison of the direction of changes in the content of selected PUFAs in the tested stool and serum samples compared to those of the control group. Thus, considering the increased content of most PUFAs in fecal samples from patients with obesity as well as the decreased serum PUFA levels reported here and in other studies, it is expected that slower digestion and/or absorption of fats rich in PUFAs may be one of the reasons for the observed phenomenon.

Another interesting observation from this study is decreased level of ECFA with 18–26 carbons in their chain, 18:1 as well as total BCFA, cyclopropane FA, and total lipids. These results may suggest more efficient lipid absorption in the intestine of subjects with obesity that may in turn contribute to morbid obesity development. Although the measurement of total fecal lipids seems a very simple analysis, we could not find any published data concerning this phenomenon. Thus, this problem requires further studies.

### Diet

Finally, a third reason for the differences in the stool LCFA profiles of patients with obesity and lean individuals may be differences in the quantity and composition of lipids consumed [30]. However, the FFQ, which was used to analyze the dietary habits of the study subjects, did not show any statistically significant differences in dietary composition between the studied groups. Both patients with morbid obesity and lean people had diets of similar quality. Therefore, we suspect that the differences in stool LCFA profiles in this study were not related to any difference in diet. However, since the evaluation of the patient’s diet was based on questionnaires, we cannot completely rule out the possibility of unidentified dietary differences between lean subjects and patients with obesity.

Additionally, both metabolic syndrome and mild hyperlipidemia found in our patients with obesity (Table 1) may contribute to alterations in the fecal contents of fatty acids.

### Consequences of Altered Fecal LCFA Profiles

The increased level of PUFAs in the stool of people with obesity may have consequences for intestinal homeostasis. First, PUFAs act on enterocyte membrane receptors, including CD36 and a number of G protein–coupled receptors (GPCRs) [30, 31] that take part in coordinating absorption and digestion and control the release of gut peptides [30]. Another consequence of the increased level of PUFAs in the stool may be an influence on the gut microbiota. It has been shown that n-3 PUFAs can change the composition of the gut microbiota [32–37]. Further studies are needed to evaluate the effects of increased PUFA content in the intestines of subjects with obesity.

The limitations of this study include a relatively small number of subjects; however, even in this groups, we were able to obtain conclusive results. Another limitation is the fact that we were not able to collect at the same time the serum and fecal samples from patients with obesity and we recruited an additional group of patients with morbid obesity to study serum fatty acid profiles. The last limitation is the use of the FFQ questionnaire to analyze the diet of subjects. Although it is not as effective tool for assessing consumption as a food diary, it allows for a quick assessment of the general eating habits of the surveyed people and the quality of their diet. Our results did not show any significant differences in the types of consumed food products between the studied groups.

## Conclusions

In conclusion, our results show for the first time that lean subjects and patients with obesity differ in their stool LCFA profiles. The levels of most n-3 PUFAs and n-6 PUFAs are significantly higher in fecal samples from people with obesity than in those from lean controls. By contrast, the levels of many PUFAs are decreased in the blood of subjects with obesity. Further studies are needed to identify the reason for this phenomenon and prove the cause-and-effect relationships, but based on our research and the data available in the literature, we can propose some potential mechanisms: alterations in fat digestion and/or LCFA absorption, and probably dietary differences that this study did not identify, or changes in enterocyte metabolism. Moreover, a very interesting finding of decreased content of total lipids in the stool of subjects with obesity warrants further studies on this subject. Changes in the fecal LCFA may affect the health status of obese subjects before and after BS. Further studies on this subject may help to develop new dietary interventions to optimize the beneficial metabolic outcomes of BS.

## Supplementary Information

Below is the link to the electronic supplementary material.

Supplementary file1 (DOCX 275 kb)

## Data Availability

All data generated or analyzed during this study are included in this published article [and its supplementary information files]. The datasets used and/or analyzed during the current study are available from the corresponding author on reasonable request.

## Abbreviations

*AdA*: Adrenic acid
*ALA*: α-Linolenic acid
*ALT*: Alanine aminotransferase
*ARA*: Arachidonic acid
*AST*: Aspartate aminotransferase
*BCFA*: Branched fatty acids
*BMI*: Body mass index
*BS*: Bariatric surgery
*COMD*: Centre of Obesity and Metabolic Diseases
*CRP*: C-reactive protein
*DGLA*: Dihomo-γ-linoleic acid
*DHA*: Docosahexaenoic acid
*DPA*: Docosapentaenoic acid
*ECFAs*: Even-chain saturated fatty acids
*EDA*: Eicosadienoic acid
*EPA*: Eicosapentaenoic acid
*FA*: Fatty acids
*FAMEs*: Fatty acid methyl esters
*FFQ-6*: Food Frequency Questionnaire-6
*GC-MS*: Gas chromatography-mass spectrometry
*GPCRs*: G protein-coupled receptors
*HDL-C*: High-density lipoprotein cholesterol
*LA*: Linoleic acid
*LC*: Lean control
*LCFA*: Long-chain fatty acid
*LDL-C*: Low-density cholesterol
*LPS*: Lipopolysaccharide
*MUFAs*: Monounsaturated fatty acids
*OCFA*: Odd-chain fatty acid
*OS*: Subjects with obesity
*PUFAs*: Polyunsaturated fatty acids
*SCFAs*: Short-chain fatty acids
*SD*: Standard deviations
*SFAs*: Saturated fatty acids
*TAG*: Triacylglycerols
*TC*: Total cholesterol
*TLR4*: Toll-like receptor 4
