# Supplementary material for: Increased Amount of Polyunsaturated Fatty Acids in the Intestinal Contents of Patients with Morbid Obesity
Source: Obes Surg. 2023 Feb 25;33(4):1228–36. doi: 10.1007/s11695-023-06518-1 (PMC10079747; doi:10.1007/s11695-023-06518-1)
Supplement: Supplementary file 1 — Supplementary file1 (DOCX 275 kb) [file 11695_2023_6518_MOESM1_ESM.docx]

SUPPLEMENTARY INFORMATION

**Increased amount of polyunsaturated fatty acids in the intestinal contents of patients with morbid obesity.**

Agata Janczy, Michał Szymański, Marta Stankiewicz, Łukasz Kaska, Ewa Stelmańska, Tomasz Śledziński, Adriana Mika

File 1.

FFQ is one of the valuable tools for assessing individual habitual dietary intake, commonly used in nutritional epidemiological studies [1]The respondents can choose from 6 categories of food consumption frequency: (1) never or almost never, (2) once a month or less frequently, (3) several times a month, (4) several times a week, (5) daily, (6) several times a day. The products presented in the survey represent 8 main food groups: sweets and snacks, dairy products and eggs, wholegrain products, fats, fruits, vegetables and seeds, meat and fish products, beverages. On the basis of the obtained data, it is possible to identify people with different levels of frequency of consumption of particular products and / or to identify characteristic patterns of food consumption.

The reproducibility of the FFQ among 20,542 participants from 123 studies was assessed in a meta-analysis by Cui et al. [2–4] The authors showed that FFQ data for most nutrients can be trusted to measure dietary intake. In addition, it is a tool that allowed to identify dietary patterns in order to find a relationship with health outcomes [2–4].

References

1. Schulz C-A, Kolade Oluwagbemigun ·, Nöthlings U. Advances in dietary pattern analysis in nutritional epidemiology. Eur J Nutr [Internet]. 2021 [cited 2022 Nov 16];60:4115–30. Available from: https://doi.org/10.1007/s00394-021-02545-9

2. Bel-Serrat S, Mouratidou T, Pala V, Huybrechts I, Börnhorst C, Fernández-Alvira JM, et al. Relative validity of the Children’s Eating Habits Questionnaire-food frequency section among young European children: the IDEFICS Study. Public Health Nutr [Internet]. Public Health Nutr; 2014 [cited 2022 Nov 16];17:266–76. Available from: https://pubmed.ncbi.nlm.nih.gov/23286734/

3. Saeedi P, Skeaff SA, Wong JE, Skidmore PML. Reproducibility and Relative Validity of a Short Food Frequency Questionnaire in 9–10 Year-Old Children. Nutrients 2016, Vol 8, Page 271 [Internet]. Multidisciplinary Digital Publishing Institute; 2016 [cited 2022 Nov 16];8:271. Available from: https://www.mdpi.com/2072-6643/8/5/271/htm

4. Lee CW, Chen HJ, Xie GR, Shih CK. Reproducibility of A Non-Quantitative Food Frequency Questionnaire (62-Item FFQ-6) and PCA-Driven Dietary Pattern Identification in 13-21-Year-Old Females. Nutrients [Internet]. Nutrients; 2019 [cited 2022 Nov 16];11. Available from: https://pubmed.ncbi.nlm.nih.gov/31514354/

**FIGURES**

**Figure S1**. Division of patients into groups along with the analyzed biological materials. F – female; M – male.

**Figure S2.** The content of bacterial fractions of fatty acids in feaces from subjects with obesity.

TABLES

**Table S1.** Fatty acids content (mg/g) in feaces from lean controls and subjects with obesity. (p.5)

**Table S2.** Selected biochemical and anthropometric characteristics in the study groups obese patients n = 23, lean subjects n = 21, not significantly different in age – p = 0.109). (p.7)

**Table S3.** The content (mg/g) of main groups of fatty acids in feaces from lean controls and subjects with obesity mathed by age (obese patients n = 23, lean subjects n = 21, not significantly different in age – p = 0.109). (p.8)

**Table S4.** The correlation coefficients between the faecal content of PUFA in obese patients (OSf) and the frequency of consumption of fat sources products. (p. 8-10)

**Table S5.** Selected biochemical and anthropometric characteristics in the study groups. (p.10)

**Table S6.** Fatty acids content (%) in serum from lean controls and obese subjects. (p. 11-12)

**Table S7.** Comparison of the direction of changes in the fatty acid content in the tested stool and serum samples compared to the control group. (p.13)


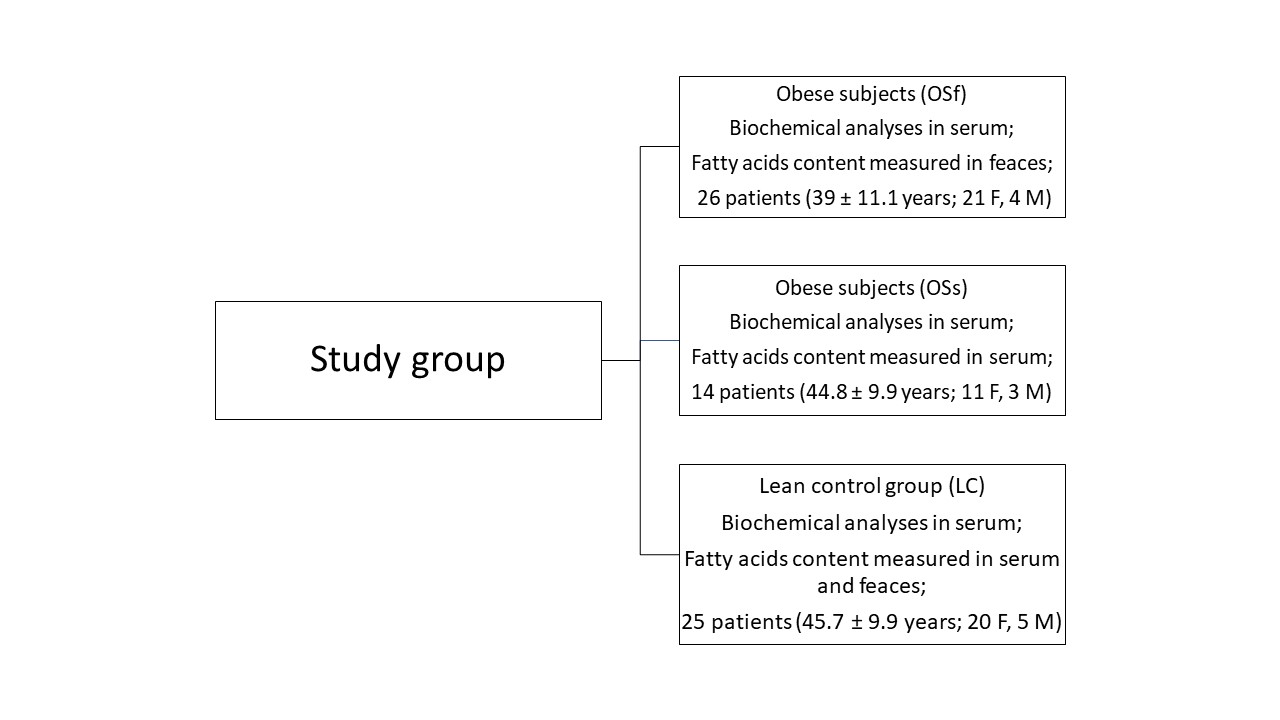


**Figure S1**. Division of patients into groups along with the analyzed biological materials. F – female; M – male.


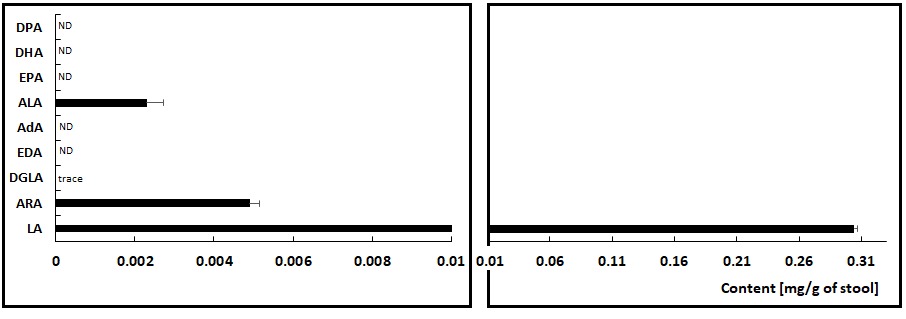


**Figure S2.** The content of bacterial fractions of fatty acids in feaces from subjects with obesity.

Data are presented as mean±SD. LA – linoleic acid, ARA – arachidonic acid, DGLA – dihomo-γ-linoleic acid, ALA - α-linolenic acid, EPA – eicosapentaenoic acid, DHA – docosahexaenoic acid, DPA – docosapentaenoic acid, ND – not detected.

**Table S1.** Fatty acids content (mg/g) in feaces from lean controls and subjects with obesity.

| **Fatty acids** | **LC** | **OSf** | **p** |
| --- | --- | --- | --- |
| 12:0 | 0.12 ± 0.36 | 0.13 ± 0.18 | 0.430 |
| 14:0 | 0.28 ± 0.29 | 0.42 ± 0.26 | **0.050** |
| 16:0 | 3.78 ± 1.58 | 4.06 ± 1.41 | 0.261 |
| 18:0 | 4.60 ± 2.19 | 3.15 ± 0.92 | **0.002** |
| 20:0 | 0.17 ± 0.09 | 0.13 ± 0.04 | **0.025** |
| 22:0 | 0.22 ± 0.09 | 0.15 ± 0.06 | **0.002** |
| 24:0 | 0.17 ± 0.08 | 0.11 ± 0.04 | **0.003** |
| 26:0 | 0.03 ± 0.03 | 0.02 ± 0.01 | **0.032** |
| 28:0 | 0.01 ± 0.01 | 0.01 ± 0.01 | 0.230 |
| 30:0 | 0.01 ± 0.01 | 0.01 ± 0.01 | 0.087 |
| 32:0 | 0.001 ± 0.001 | 0.001 ± 0.001 | 0.243 |
| **Total ECFA** | **9.40 ± 3.19** | **8.20 ± 2.26** | 0.073 |
| 13:0 | 0.004 ± 0.003 | 0.005 ± 0.003 | 0.470 |
| 15:0 | 0.24 ± 0.14 | 0.16 ± 0.10 | **0.021** |
| 17:0 | 0.09 ± 0.05 | 0.10 ± 0.05 | 0.210 |
| 19:0 | 0.01 ± 0.01 | 0.01 ± 0.01 | 0.155 |
| 21:0 | 0.01 ± 0.00 | 0.01 ± 0.00 | 0.458 |
| 23:0 | 0.04 ± 0.02 | 0.04 ± 0.02 | 0.391 |
| 25:0 | 0.01 ± 0.01 | 0.01 ± 0.00 | 0.117 |
| 27:0 | 0.004 ± 0.004 | 0.003 ± 0.002 | 0.083 |
| **Total OCFA** | **0.41 ± 0.21** | **0.34 ± 0.17** | 0.098 |
| *anteiso* 10-methyl-12:0 | 0.003 ± 0.002 | 0.002 ± 0.001 | 0.199 |
| *anteiso* 12- methyl-14:0 | 0.22 ± 0.13 | 0.18 ± 0.10 | 0.158 |
| anteiso 14-methyl-16:0 | 0.03 ± 0.02 | 0.03 ± 0.02 | 0.187 |
| anteiso 16-methyl-18:0 | 0.01 ± 0.00 | 0.01 ± 0.00 | 0.403 |
| anteiso 20-methyl-22:0 | 0.002 ± 0.002 | 0.001 ± 0.000 | 0.085 |
| **Total *anteiso* BCFA** | **0.26 ± 0.15** | **0.22 ± 0.12** | 0.149 |
| *iso* 11-methyl-12:0 | 0.003 ± 0.003 | 0.002 ± 0.002 | 0.204 |
| *iso* 12-methyl-13:0 | 0.01 ± 0.01 | 0.01 ± 0.01 | 0.126 |
| *iso* 13-methyl-14:0 | 0.08 ± 0.05 | 0.05 ± 0.03 | **0.030** |
| *iso* 14-methyl-15:0 | 0.06 ± 0.05 | 0.03 ± 0.03 | **0.002** |
| *iso* 15-methyl-16:0 | 0.06 ± 0.05 | 0.04 ± 0.03 | **0.038** |
| *iso* 16-methyl-17:0 | 0.02 ± 0.01 | 0.01 ± 0.01 | **0.022** |
| *iso* 20-methyl-21:0 | 0.001 ± 0.001 | 0.001 ± 0.001 | 0.161 |
| *iso* 22-methyl-23:0 | 0.002 ± 0.002 | 0.001 ± 0.001 | 1.000 |
| **Total *iso* BCFA** | **0.24 ± 0.14** | **0.14 ± 0.08** | **0.004** |
| 2,6,10-trimethyl-12:0 | 0.003 ± 0.006 | 0.002 ± 0.002 | 0.086 |
| 4,8,12-trimethyl-14:0 | 0.003 ± 0.002 | 0.002 ± 0.002 | 0.061 |
| **Total tri-methyl -BCFA** | **0.01 ± 0.01** | **0.00 ± 0.00** | **0.032** |
| **Total BCFA** | **0.56 ± 0.28** | **0.39 ± 0.20** | **0.010** |
| **Total SFA** | **10.4 ± 3.53** | **8.93 ± 2.46** | 0.056 |
| 14:1 | 0.00 ± 0.00 | 0.01 ± 0.01 | **0.034** |
| 15:1 | 0.005 ± 0.004 | 0.005 ± 0.007 | 0.439 |
| 16:1 | 0.16 ± 0.08 | 0.16 ± 0.07 | 0.477 |
| 17:1 | 0.02 ± 0.02 | 0.01 ± 0.01 | 0.181 |
| 18:1 | 6.44 ± 2.13 | 4.37 ± 1.48 | **0.001** |
| 19:1 | 0.002 ± 0.001 | 0.002 ± 0.001 | 0.230 |
| 20:1 | 0.06 ± 0.06 | 0.05 ± 0.04 | 0.296 |
| 22:1 | 0.02 ± 0.03 | 0.02 ± 0.02 | 0.402 |
| 24:1 | 0.03 ± 0.03 | 0.03 ± 0.02 | 0.418 |
| **Total MUFA** | **6.74 ± 2.11** | **4.66 ± 1.47** | **0.001** |
| **Total Cyclopropane FA** | **0.03 ± 0.02** | **0.02 ± 0.01** | **0.036** |
| 18:3 ALA | 0.012 ± 0.010 | 0.002 ± 0.002 | **0.003** |
| 20:5 EPA | 0.002 ± 0.004 | 0.005 ± 0.007 | 0.103 |
| 22:5 DPA | 0.003 ± 0.004 | 0.011 ± 0.016 | **0.019** |
| 22:6 DHA | 0.003 ± 0.003 | 0.007 ± 0.009 | **0.031** |
| **Total n-3 PUFA** | **0.02 ± 0.02** | **0.03 ± 0.03** | **0.243** |
| 18:2 LA | 2.82 ± 2.32 | 1.80 ± 1.56 | **0.04** |
| 20:3 DGLA | 0.00 ± 0.01 | 0.03 ± 0.05 | **0.01** |
| 20:4 ARA | 0.01 ± 0.01 | 0.03 ± 0.04 | **0.00** |
| 20:2 n-6 | 0.00 ± 0.00 | 0.01 ± 0.02 | **0.00** |
| 22:4 n-6 | 0.002 ± 0.002 | 0.007 ± 0.009 | **0.00** |
| **Total n-6 PUFA** | **2.84 ± 2.31** | **1.88 ± 1.54** | **0.05** |
| **Total LCFA** | **2.88 ± 2.31** | **1.93 ± 1.53** | **0.051** |

Data are presented as mean ± SD. Significant difference compared with healthy controls at P <0.05. ECFA – even chain saturated fatty acids; OCFA – odd-chain fatty acids; BCFA – branched chain fatty acids; SFA – saturated fatty acids; MUFA – monounsaturated fatty acids; Cyclopropane FA – cyclopropane fatty acids; ALA - α-linolenic acid; EPA – eicosapentaenoic acid; DPA – docosapentaenoic acid; DHA – docosahexaenoic acid; PUFA – polyunsaturated fatty acid; LA – linoleic acid; DGLA – dihomo-γ-linoleic acid; LCFA – long chain fatty acids.

**Table S2.** Selected biochemical and anthropometric characteristics in the study groups obese patients n = 23, lean subjects n = 21, not significantly different in age – p = 0.109).

| **Parameter** | **LC** | **OSf** |
| --- | --- | --- |
| **Age (years)** | 40.6 ± 7.55 | 43.9 ± 8.97 |
| **BMI (kg/m^2^)** | 22.7 ± 2.44 | 40.7 ± 5.83^#^ |
| **TC (mg/dl)** | 186 ± 30.6 | 206 ± 43.0 |
| **HDL-C (mg/dl)** | 64.2 ± 8.93 | 52.7 ± 11.7* |
| **LDL-C (mg/dl)** | 103 ± 23.2 | 122 ± 34.7* |
| **TAG (mg/dl)** | 92.4 ± 45.3 | 133 ± 81.5^#^ |
| **ALT (U/l)** | ND | 36.6 ± 35.5 |
| **AST (U/l)** | ND | 24.4 ± 13.5 |
| **Glucose (mg/dl)** | 87.1 ± 3.82 | 93.5 ± 9.11* |
| **Insulin (mU/ml)** | 6.66 ± 2.12 | 12.7 ± 7.69^#^ |
| **HOMA-IR** | 1.44 ± 0.50 | 2.93 ± 2.16* |
| **CRP (mg/l)** | 1.74 ± 1.21 | 7.45 ± 5.91^#^ |
| **Albumin (g/l)** | 38.9 ± 2.15 | 40.6 ± 2.98 |
| **Interleukin 6 (pg/ml)** | ND | 3.10 ± 0.76 |

Data are presented as mean ± SD. LC – lean controls, OSf – obese subjects with obesity (group included in analysis of feaces), BMI – body mass index, TC – total cholesterol, HDL-C – high-density cholesterol, LDL-C – low-density cholesterol, TAG – triacylglycerols, ALT – alanine aminotransferase, AST – aspartate aminotransferase, CRP – C-reactive protein, ND – not determined.

* Significant difference compared with healthy controls at P <0.05; # Significant difference compared with healthy controls at P <0.001

**Table S3. The content (mg/g) of main groups of fatty acids in feaces from lean controls and subjects with obesity mathed by age (obese patients n = 23, lean subjects n = 21, not significantly different in age – p = 0.109).**

| **Fatty acids** | **LC** | **OSf** | **p** |
| --- | --- | --- | --- |
| **Total ECFA** | 9.34 ± 3.34 | 8.20 ± 2.26 | 0.094 |
| **Total OCFA** | 0.31 ± 0.21 | 0.34 ± 0.17 | 0.162 |
| **Total BCFA** | 0.53 ± 0.27 | 0.39 ± 0.20 | 0.031 |
| **Total SFA** | 10.3 ± 3.67 | 8.93 ± 2.46 | 0.080 |
| **Total MUFA** | 6.69 ± 2.07 | 4.66 ± 1.47 | **0.000** |
| **Total Cyclopropane FA** | 0.02 ± 0.01 | 0.02 ± 0.01 | 0.071 |
| **Total n-3 PUFA** | 0.02 ± 0.02 | 0.03 ± 0.03 | 0.212 |
| **Total n-6 PUFA** | 3.03 ± 2.40 | 1.89 ± 1.54 | **0.031** |
| **Total LCFA** | 3.07 ± 2.43 | 1.93 ± 1.59 | 0.679 |

Data are presented as mean ± SD. Significant difference compared with healthy controls at P <0.05. ECFA – even chain saturated fatty acids; OCFA – odd-chain fatty acids; BCFA – branched chain fatty acids; SFA – saturated fatty acids; MUFA – monounsaturated fatty acids; Cyclopropane FA – cyclopropane fatty acids; PUFA – polyunsaturated fatty acid; LCFA – long chain fatty acids.

**Table S4.** **The correlation coefficients between the faecal content of PUFA in obese patients (OSf) and the frequency of consumption of fat sources products.**

The composition of the subjects OSf and LC diet were assessed based of the FFQ-6 questionnaires. In the OSf group, products that are a source of fat in the diet were selected from the results of the FFQ questionnaire analyzes. Spearman's test showed several significant correlations between the consumption of selected sources of fat in the diet with individual n-3 and n-6 PUFAs in the stool of the OSf group.

|  | **ALA** | **EPA** | **DHA** | **DPA** | **PUFA n-3** | **LA** | **ARA** | **DGLA** | **20:2 n-6** | **22:4 n-6** | **PUFA n-6** | **PUFA OTHER** |
| --- | --- | --- | --- | --- | --- | --- | --- | --- | --- | --- | --- | --- |
| **Chocolates** | -0.006 | -0.089 | -0.388 | -0.136 | -0.163 | 0.171 | 0.039 | -0.042 | 0.071 | 0.059 | 0.187 | 0.016 |
| **Biscuits and cakes** | 0.013 | -0.187 | -0.369 | -0.243 | -0.252 | 0.087 | -0.034 | -0.209 | 0.058 | -0.183 | 0.083 | -0.228 |
| **Ice creams** | -0.309 | 0.073 | -0.244 | -0.080 | -0.140 | 0.377 | 0.158 | 0.117 | 0.195 | 0.082 | 0.374 | 0.130 |
| **Salty snakcks** | 0.036 | -0.305 | -0.350 | -0.265 | -0.206 | -0.288 | -0.275 | -0.203 | -0.208 | -0.253 | -0.296 | 0.114 |
| **Milk and milk drinks** | 0.224 | 0.139 | 0.351 | 0.311 | 0.251 | -0.256 | 0.150 | 0.246 | -0.258 | 0.342 | -0.215 | -0.005 |
| **Sweetened milk drinks** | -0.358 | 0.185 | -0.070 | 0.012 | -0.009 | -0.170 | 0.205 | 0.153 | 0.184 | -0.021 | -0.170 | -0.125 |
| **Natural curds** | 0.121 | -0.189 | 0.297 | 0.050 | -0.023 | 0.210 | 0.125 | 0.112 | -0.063 | 0.139 | 0.210 | 0.034 |
| **Flavored cottage cheese** | -0.080 | -0.239 | -0.109 | -0.195 | -0.252 | 0.113 | 0.128 | -0.059 | 0.130 | -0.048 | 0.101 | 0.116 |
| **Cheese** | -0.240 | 0.019 | 0.119 | 0.112 | 0.078 | -0.193 | 0.052 | 0.263 | -0.112 | 0.092 | -0.169 | 0.137 |
| **Eggs and eggs dishes** | 0.260 | -0.501 | -0.199 | -0.287 | -0.270 | 0.233 | -0.472 | -0.507 | -0.340 | -0.216 | 0.243 | -0.278 |
| **Whole meal bread** | -0.394 | -0.106 | -0.167 | -0.281 | -0.299 | 0.221 | -0.038 | -0.221 | 0.225 | -0.283 | 0.196 | -0.169 |
| **White bread** | -0.103 | 0.080 | -0.144 | 0.168 | 0.108 | -0.177 | -0.073 | 0.142 | -0.170 | 0.213 | -0.166 | -0.140 |
| **Ready-made cereal products** | -0.337 | 0.016 | 0.344 | -0.006 | -0.037 | 0.095 | 0.346 | 0.266 | 0.159 | 0.142 | 0.125 | 0.068 |
| **Oil** | 0.167 | 0.072 | -0.233 | 0.254 | 0.234 | -0.485 | 0.110 | 0.265 | -0.103 | 0.246 | -0.455 | -0.057 |
| **Butter** | -0.260 | -0.051 | -0.323 | -0.060 | -0.078 | 0.030 | 0.247 | 0.022 | 0.351 | -0.042 | 0.046 | 0.110 |
| **Margarine** | -0.470 | -0.078 | -0.301 | -0.302 | -0.326 | -0.036 | -0.176 | -0.191 | 0.114 | -0.397 | -0.049 | 0.090 |
| **Sour cream** | -0.022 | -0.186 | -0.098 | 0.037 | -0.023 | -0.096 | 0.059 | -0.007 | 0.050 | 0.148 | -0.088 | 0.283 |
| **Lard** | 0.289 | 0.081 | -0.070 | 0.140 | 0.177 | 0.041 | 0.126 | -0.054 | 0.016 | -0.007 | 0.051 | -0.044 |
| **Mayonnaise** | 0.332 | 0.002 | -0.152 | 0.130 | 0.174 | -0.413 | -0.002 | -0.002 | -0.163 | 0.188 | -0.387 | 0.031 |
| **Avocado** | 0.483 | 0.148 | 0.370 | 0.319 | 0.342 | 0.113 | 0.159 | 0.153 | -0.211 | 0.245 | 0.136 | 0.045 |
| **Olives** | 0.306 | -0.090 | -0.033 | 0.200 | 0.192 | 0.023 | 0.170 | 0.099 | -0.078 | 0.167 | 0.036 | 0.075 |
| **Nuts** | -0.148 | -0.202 | 0.220 | 0.083 | 0.044 | 0.047 | 0.309 | 0.363 | 0.198 | 0.228 | 0.041 | -0.011 |
| **Seeds** | 0.225 | -0.099 | 0.105 | -0.077 | -0.011 | 0.461 | -0.050 | -0.162 | -0.029 | -0.082 | 0.465 | 0.217 |
| **Sausages** | 0.174 | -0.088 | -0.219 | 0.041 | 0.026 | 0.010 | 0.042 | -0.092 | -0.064 | -0.100 | 0.042 | -0.138 |
| **High-quality meats/loin** | -0.042 | -0.080 | -0.363 | 0.026 | -0.048 | -0.055 | -0.014 | 0.049 | -0.107 | 0.154 | -0.045 | 0.161 |
| **Meat offal** | 0.100 | -0.008 | -0.346 | -0.101 | -0.079 | 0.286 | 0.015 | -0.225 | 0.019 | -0.153 | 0.288 | 0.036 |
| **Red meat** | 0.041 | 0.035 | -0.018 | 0.144 | 0.148 | -0.062 | 0.379 | 0.188 | 0.256 | 0.008 | -0.039 | -0.135 |
| **Poultry/rabbit** | -0.147 | -0.118 | -0.180 | 0.027 | -0.113 | 0.133 | 0.010 | 0.104 | 0.052 | 0.198 | 0.118 | 0.177 |
| **Venison** | 0.217 | -0.173 | -0.160 | -0.204 | -0.156 | 0.238 | -0.188 | -0.377 | -0.223 | -0.252 | 0.238 | -0.400 |
| **Lean fish** | 0.191 | -0.286 | -0.118 | 0.116 | 0.055 | 0.097 | -0.019 | 0.022 | 0.106 | 0.232 | 0.084 | -0.134 |
| **Fatty fish** | 0.156 | -0.408 | -0.090 | 0.032 | -0.093 | 0.105 | -0.060 | -0.014 | 0.004 | 0.336 | 0.105 | 0.179 |

Positive correlations are marked in green, negative in red. ALA - α-linolenic acid, EPA – eicosapentaenoic acid, DPA – docosapentaenoic acid, DHA – docosahexaenoic acid, PUFA – polyunsaturated fatty acid, LA – linoleic acid, DGLA – dihomo-γ-linoleic acid, ARA – arachidonic acid.

**Table S5.** Selected biochemical and anthropometric characteristics in the study groups.

| **Parameter** | **LC** | **OSs** |
| --- | --- | --- |
| **Age (years)** | 45.7 ± 9.93 | 44.6 ± 9.99 |
| **BMI (kg/m^2^)** | 23.2 ± 2.33 | 37.6 ± 1.88^#^ |
| **TC (mg/dl)** | 198 ± 31.4 | 161 ± 32.3^#^ |
| **HDL-C (mg/dl)** | 59.4 ± 12.1 | 38.5 ± 5.37^#^ |
| **LDL-C (mg/dl)** | 116 ± 31.3 | 106 ± 32.2 |
| **TAG (mg/dl)** | 113 ± 67.5 | 108 ± 30.5 |
| **ALT (U/l)** | ND | 56.0 ± 19.8 |
| **AST (U/l)** | ND | 30.5 ± 3.53 |
| **Glucose (mg/dl)** | 87.2 ± 6.26 | 116 ± 38.4* |
| **Insulin (mU/ml)** | 7.14 ± 2.56 | 18.9 ± 14.5^#^ |
| **HOMA-IR** | 1.54 ± 0.55 | 4.50 ± 2.55 |
| **CRP (mg/l)** | 1.56 ± 1.11 | 7.24 ± 7.86^#^ |
| **Albumin (g/l)** | 39.3 ± 1.98 | 38.2 ± 1.5 |

Data are presented as mean ± SD. LC – lean controls, OSs –subjects with obesity (serum group), BMI – body mass index, TC – total cholesterol, HDL-C – high-density cholesterol, LDL-C – low-density cholesterol, TAG – triacylglycerols, ALT – alanine aminotransferase, AST – aspartate aminotransferase, CRP – C-reactive protein, ND – not determined. * Significant difference compared with healthy controls at P <0.05; ^#^ Significant difference compared with healthy controls at P <0.001.

**Table S6.** **Fatty acids content (%) in serum from lean controls and obese subjects.**

The results of subjects with obesity were compared to lean controls by Student’s t-test. The number of studied samples in FA assays: 14 subjects with obesity and 25 lean subjects. Values are the mean ± SD.

| **Fatty acids** | **LC** | **OSs** | **p** |
| --- | --- | --- | --- |
| 12:0 | 0.22 ± 0.08 | 0.19 ± 0.08 | 0.324 |
| 14:0 | 1.24 ± 0.29 | 1.16 ± 0.34 | 0.491 |
| 16:0 | 23.6 ± 1.93 | 24.5 ± 0.91 | 0.123 |
| 18:0 | 7.27 ± 0.75 | 6.27 ± 0.53 | **<0.001** |
| 20:0 | 0.08 ± 0.02 | 0.13 ± 0.04 | **<0.001** |
| 22:0 | 0.15 ± 0.04 | 0.23 ± 0.12 | **0.037** |
| 24:0 | 0.15 ± 0.05 | 0.19 ± 0.11 | 0.158 |
| **Total ECFA** | **32.7 ± 1.94** | **32.6 ± 0.63** | 0.924 |
| 13:0 | 0.03 ± 0.01 | 0.02 ± 0.01 | **0.029** |
| 15:0 | 0.24 ± 0.05 | 0.27 ± 0.07 | 0.184 |
| 17:0 | 0.26 ± 0.05 | 0.26 ± 0.02 | 0.876 |
| 19:0 | 0.03 ± 0.01 | 0.02 ± 0.01 | 0.123 |
| 21:0 | 0.02 ± 0.01 | 0.04 ± 0.04 | **0.045** |
| 23:0 | 0.06 ± 0.02 | 0.09 ± 0.05 | 0.111 |
| **Total OCFA** | **0.65 ± 0.12** | **0.70 ± 0.12** | 0.232 |
| 4,8,12-methyl-14:0 | 0.01 ± 0.00 | 0.02 ± 0.01 | **0.020** |
| *anteiso* 12-methyl-14:0 | 0.03 ± 0.01 | 0.05 ± 0.03 | **0.049** |
| *anteiso* 14-methyl-16:0 | 0.07 ± 0.02 | 0.12 ± 0.07 | **0.025** |
| *anteiso* 16-methyl-18:0 | 0.09 ± 0.04 | 0.04 ± 0.01 | **<0.001** |
| *anteiso* 20-methyl-22:0 | 0.01 ± 0.00 | 0.02 ± 0.01 | **0.004** |
| **Total *anteiso* BCFA** | **0.21 ± 0.04** | **0.23 ± 0.09** | 0.409 |
| *iso* 12-methyl-13:0 | 0.01 ± 0.01 | 0.01 ± 0.01 | 0.564 |
| *iso* 13-methyl-14:0 | 0.03 ± 0.01 | 0.03 ± 0.01 | **0.034** |
| *iso* 14-methyl-15:0 | 0.07 ± 0.02 | 0.05 ± 0.01 | **<0.001** |
| *iso* 15-methyl-16:0 | 0.09 ± 0.04 | 0.08 ± 0.02 | 0.213 |
| *iso* 21-methyl-21:0 | 0.01 ± 0.00 | 0.01 ± 0.01 | 0.438 |
| **Total  *iso* BCFA** | **0.21 ± 0.05** | **0.17 ± 0.02** | **0.036** |
| **Total BCFA** | **0.43 ± 0.09** | **0.41 ± 0.12** | 0.678 |
| **Total SFA** | **33.8 ± 1.80** | **33.8 ± 0.59** | 0.979 |
| 14:1 | 0.08 ± 0.03 | 0.07 ± 0.04 | 0.198 |
| 16:1 | 3.09 ± 0.95 | 3.68 ± 1.19 | 0.160 |
| 18:1 | 26.1 ± 3.15 | 29.1 ± 2.76 | **0.015** |
| 19:1 | 0.03 ± 0.01 | 0.02 ± 0.01 | 0.277 |
| 20:1 | 0.18 ± 0.04 | 0.17 ± 0.03 | 0.162 |
| 22:1 | 0.05 ± 0.03 | 0.03 ± 0.01 | **0.037** |
| 24:1 | 0.26 ± 0.12 | 0.30 ± 0.13 | 0.352 |
| **Total MUFA** | **32.9 ± 4.45** | **33.3 ± 3.35** | 0.787 |
| **Total cyklopropane FA** | **0.17 ± 0.03** | **0.16 ± 0.04** | 0.510 |
| **Total LCFA** | **36.3 ± 4.69** | **32.7 ± 3.48** | **0.016** |

Data are presented as mean ± SD. Significant difference compared with healthy controls at P <0.05. ECFA – even chain saturated fatty acids; OCFA – odd-chain fatty acids; BCFA – branched chain fatty acids; SFA – saturated fatty acids; MUFA – monounsaturated fatty acids; Cyclopropane FA – cyclopropane fatty acids; LCFA – long chain fatty acids.

**Table S7.** **Comparison of the direction of changes in the fatty acid content in the tested stool and serum samples compared to the control group.**


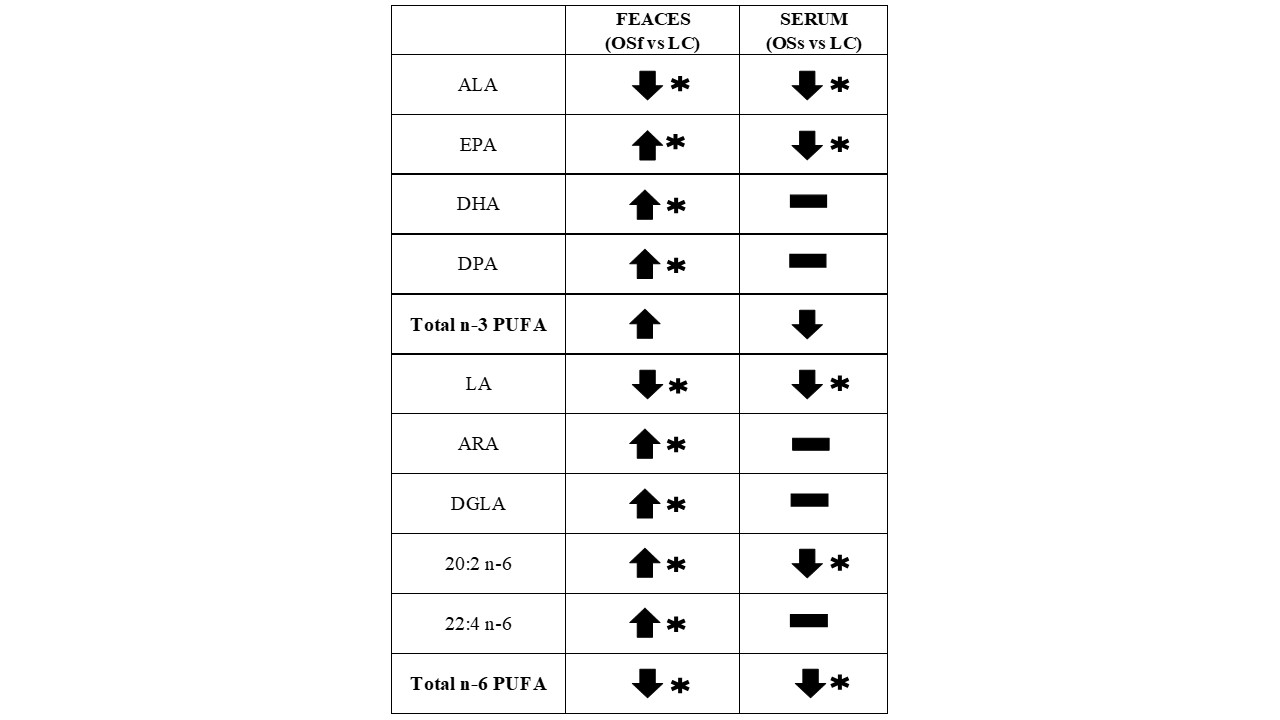


*Data are statistically significant (p<0.05). Arrows without * represents the trends. – represent no change
